# Determinants of between-year burrow re-occupation in a colony of the European bee-eater *Merops apiaster*

**DOI:** 10.1002/ece3.1563

**Published:** 2015-07-15

**Authors:** Vera Brust, Hans-Valentin Bastian, Anita Bastian, Tim Schmoll

**Affiliations:** 1 Department of Behavioural Biology, University of OsnabrueckBarbarastraße 11, 49076, Osnabrueck, Germany; 2 Geschwister-Scholl-Str. 15, 67304Kerzenheim, Germany; 3 Evolutionary Biology, Bielefeld UniversityMorgenbreede 45, 33615, Bielefeld, Germany

**Keywords:** Breeding ecology, breeding philopatry, burrow re-occupation, burrow reuse, European bee-eater, *Merops apiaster*

## Abstract

Re-occupation of existing nesting burrows in the European bee-eater *Merops apiaster* has only rarely – and if so mostly anecdotically – been documented in the literature record, although such behavior would substantially save time and energy. In this study, we quantify burrow re-occupation in a German colony over a period of eleven years and identify ecological variables determining reuse probability. Of 179 recorded broods, 54% took place in a reused burrow and the overall probability that one of 75 individually recognized burrows would be reused in a given subsequent year was estimated as 26.4%. This indicates that between-year burrow reuse is a common behavior in the study colony which contrasts with findings from studies in other colonies. Furthermore, burrow re-occupation probability declined highly significantly with increasing age of the breeding wall. Statistical separation of within- and between-burrow effects of the age of the breeding wall revealed that a decline in re-occupation probability with individual burrow age was responsible for this and not a selective disappearance of burrows with high re-occupation probability over time. Limited duty cycles of individual burrows may be caused by accumulating detritus or decreasing stability with increasing burrow age. Alternatively, burrow fidelity may presuppose pair fidelity which may also explain the observed restricted burrow reuse duty cycles. A consequent next step would be to extend our within-colony approach to other colonies and compare the ecological circumstances under which bee-eaters reuse breeding burrows.

## Introduction

**Figure 1:**
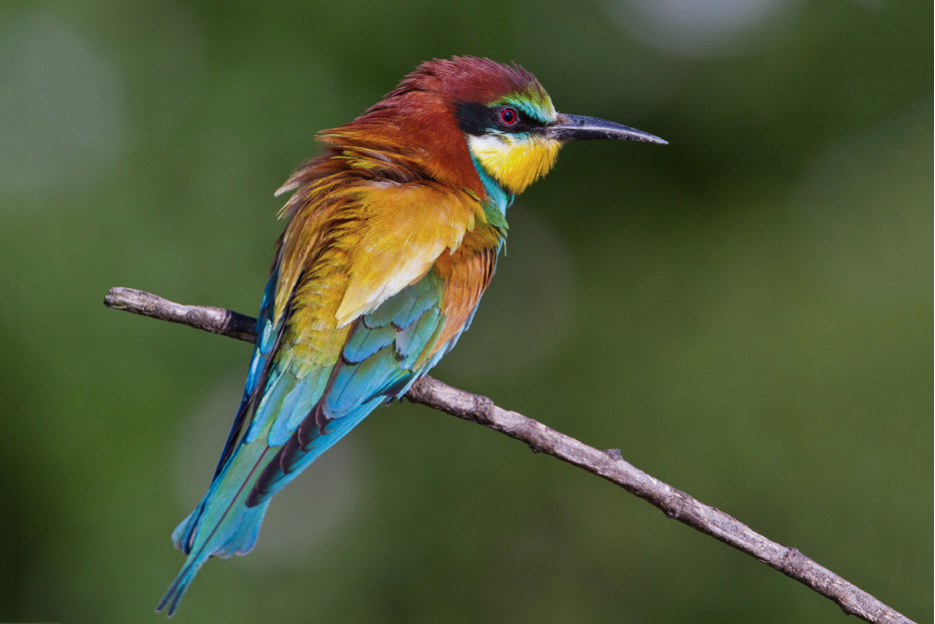
European bee-eater *Merops apiaster* at a southern German breeding wall. Photograph credit: Uwe Nielsen.

The European bee-eater (*Merops apiaster*) (Fig.1) is a widely distributed gregarious bird species that breeds colonially mainly in Europe, northwestern Africa, the Caucasus, western Russia, and Central and Southwest Asia (BirdLife International, 2004). Bee-eaters are socially and seasonally monogamous and lay on average 5–7 eggs in a single clutch per year (Glutz von Blotzheim and Bauer 1994). Eggs are deposited in nesting chambers excavated at the end of 70- to 210-cm-long tunnels, mainly in vertical or at least very strongly sloped cliffs (Glutz von Blotzheim and Bauer 1994) composed of sandy soils of a grit size of 20–100 *μ*m (Heneberg and Šimeček 2004). Partners excavate burrows together over six to twelve days (Hahn 1981), taking turns in resting and burrowing. Depending on the soil composition, each pair of birds thus has to remove between 2.1 and 8.8 L of soil to build a burrow, which corresponds to 5 – 13 kg of soil that has to be moved (White et al. 1977; Casas-Crivillé and Valera 2005).

Bee-eaters regularly use the same breeding walls over several years (Todte et al. 1999; Arbeiter et al. 2012; Bastian et al. 2013), and Peters and Trapp (2012) demonstrated individual breeding philopatry, that is, the repeated use of the same wall for breeding in 22% of 87 individually marked birds. Even if breeding philopatry is rather moderate in European bee-eaters, a reuse of existing nesting burrows across years clearly comprises the potential benefit to save time and energy. Both, clay sand as well as solid loess substrate, which are predominately used for burrow construction (Heneberg and Šimeček 2004; Bastian et al. 2013; McLaren et al. 2014) are suggested to keep the burrows in potentially good working condition over years which may facilitate reuse. Reusing old burrows often holds the advantage of an advanced laying date and energy saving. In a number of facultative burrow reusing species such as chickadees, nuthatches, and woodpeckers, larger clutch sizes, better provisioning of offspring, and consequently a higher recruitment in individuals that bred in already existing burrows compared to conspecifics that excavated a new burrow were found (Wiebe et al. 2007) (Fig.1).

Bee-eaters do not use conventional nesting materials but lay their eggs on the bare soil of the burrow. However, in the burrow they regularly regurgitate indigestible stomach contents, mostly consisting of chitin remains of their insect diet, which serves as some kind of bedding for the eggs (Glutz von Blotzheim and Bauer 1994). Within these pellets, large numbers of living larvae of flies, beetles, and other insects have been found (Ursprung 1984). Aside from the pellets, the droppings of nestlings are not removed from the burrow by the parents and seep into the chitin deposits. Additionally, removal of unhatched eggs or dead nestlings has also not been documented in the species (Krimmer and Piechocki 1974; Glutz von Blotzheim and Bauer 1994). Taken together, any reuse of burrows might increase the risk of diseases and parasite infection which might select for constructing burrows anew on a yearly basis.

In line with this reasoning, obligate new construction of breeding burrows each year is reported for the European bee-eater from a number of sources (Ursprung 1984; Glutz von Blotzheim and Bauer 1994; Todte et al. 1999). While to our knowledge quantitative evidence to support the claim of obligate new construction is lacking in the European bee-eater, a systematic field study conducted on the closely related Rainbow bee-eater (*Merops ornatus*) indeed found no evidence for any burrow reuse (Boland 2004). Fry (2001) even generalizes over the whole family of *Meropidae* that nearly always burrows are excavated anew each year and reuse of the previous year’s nests is rare. In the European bee-eater, only very few sources report that burrow reuse occurs at all and if so that it is rare (Cramp 1981; Krištín 1992; Rupp and Saumer 1996; Casas-Crivillé and Valera 2005; Rupp et al. 2011). From these studies, only Rupp and Saumer (1996) and Rupp et al. (2011) provide some quantitative data. For the Southern upper Rhine valley, they report that 19 of 134 broods (14%) took place in previously used breeding burrows between 1990 and 1996 and that individual burrows were used up to four times although not necessarily in consecutive years.

In this study, we investigate between-year burrow reuse and its dynamics in a European bee-eater colony in southern Germany across a period of eleven years. We document that, in contrast to what is reported in the literature, between-year burrow reuse is common in this study colony. Furthermore, we test competing hypotheses that could explain mechanistically an observed general decline in re-occupation probability with increasing age of the breeding wall by statistically separating within- from between-burrow effects. More precisely, we test the hypothesis that (1) a decline in re-occupation probability is due to a limited duty cycle of individual burrows, for example caused by increasing contamination with detritus or parasites or a decrease in burrow stability over time (*burrow age hypothesis*); or alternatively (2) a decline in re-occupation probability is due to the fact that during colony establishment, unconstrained construction of high-quality burrows is possible, while in later years, burrows are built increasingly in suboptimal parts of the breeding wall with regard to, for example, soil properties or predation risk (*burrow quality hypothesis*).

## Methods

### Study colony and field methods

The study colony is located near Eisenberg, Rhineland-Palatinate, Germany, in the “Alzeyer Hügelland” where we observed the birds at an approximately 55 m² clay sand wall that is part of a commercially exploited sand pit, which exists much longer than the birds actually started to use it as a breeding wall in 2003 (Bastian and Bastian 2003). Since then, detailed behavioral observations have been made at the focal wall every year until 2013 from a distance of at least 50 m using binoculars. With few exceptions, short checks were carried out on a daily basis from the end of April/beginning of May (depending on the arrival time of the birds in the respective year) to the end of May, continued later by weekly observations until the birds left the breeding colony. Observations were usually made between morning and noon or in the late afternoon during the main flight times of insects that serve as the bee-eater’s diet and consequently their peak activity (Inglisa and Galeotti 1993). We classified a burrow as occupied in a given year when fledglings were observed in it, which was the case in roughly 90% of the 179 recorded successful broods, or if adult birds were observed to enter it at least three times within a 2-week period while simultaneously carrying food on at least one of these occasions. Based on these criteria, we could be certain that a breeding burrow labeled as occupied was indeed a completely excavated tunnel including a nest chamber and chicks. For burrows that were already occupied in a previous year, we probed re-occupation in subsequent years applying the same criterion. Burrows could be individually identified over the whole study period by photographic surveys documented each year.

Besides a single year in which two pairs of sand martins could also be observed in the breeding wall, bee-eaters were the only burrowing birds to use the wall under study. For this study, no intervention with birds or nesting burrows took place. Thus, we do not have any information on the interior conditions of the burrows, for example, whether the nesting chamber has collapsed or if the bee-eaters, such as sand martins, enlarge a reused burrow by adding a new nest chamber (Stoner and Stoner 1941; Kuhnen 1975). As birds were not marked individually, no information regarding pair identity or fledgling/recruit survival was available. For the photographic surveys, pictures had to be taken from different angles due to changing working situations in the active sand pit. Consequently, no information about the absolute position of burrows in the wall could be retrieved from this data.

### Statistical analysis

We tested for the fixed effect of the *age of the breeding wall* on between-year burrow re-occupation probability using generalized linear mixed models (GLMM) with binomial error structure and logit link function. We transformed the original covariate *age of the breeding wall* (ranging from 1 to 10) by subtracting 5.5 such that (biologically sensible) estimates for the intercepts in our models are given for the median age of the breeding wall (instead for a breeding wall age of zero years). We included study year and breeding burrow identity as random intercept effects as well as random slope effects for *age of the breeding wall* on breeding burrow identity in our models to control for pseudoreplication due to the lack of independence resulting from multiple observations of the same burrow or from the same year. We refrained from including in addition a random slope effect for *age of the breeding wall* on study year due to the observed lack of convergence when fitting such models. We estimated the overall re-occupation probability using a binomial GLMM with the intercept as only fixed effect and burrow identity and study year as random intercept effects to obtain an unbiased estimate for overall mean re-occupation probability (including appropriate standard errors) which is controlled for pseudoreplication.

**Figure 2:**
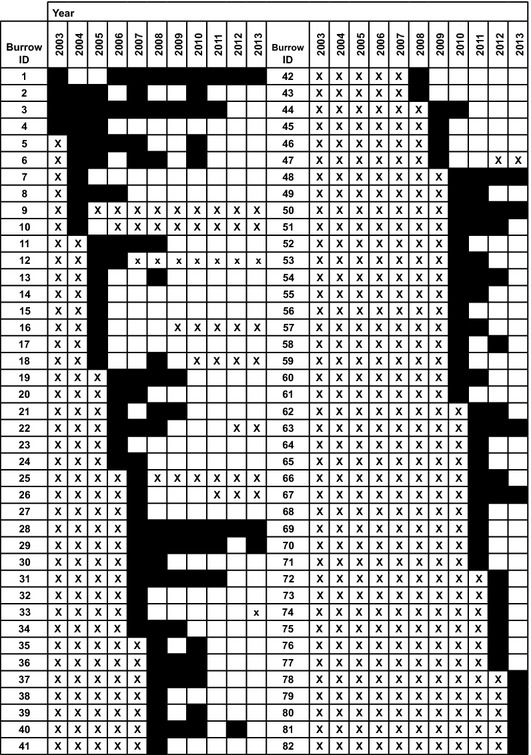
Patterns of between-year (re-)occupation of 82 individually recognized nesting burrows in a southern German colony of the European bee-eater *Merops apiaster*. Black coloration denotes occupation, white coloration nonoccupation, and *X* that a burrow had not yet or not any more existed in the respective year. Black and white colored cells sum up to a total of 424 burrow-year observations made across the study period.

Not all the burrows could be monitored across the entire study period due to first appearance of burrows after establishment of the breeding wall (in 2003) and/or disappearance before the end of the observation period (in 2013, see Fig.2). We therefore applied within-subject (i.e. within-burrow) centering of covariates in additional regression models to tease apart within- from between-burrow effects. Within-burrow centering allows distinguishing whether any significant effects result from changes in re-occupation probability of individual burrows or rather from the selective (dis-)appearance of burrows with particular re-occupation probabilities (or a combination of both, see van de Pol and Wright 2009 for details). Note that the *within-burrow age of the breeding wall* effect precisely models the age of individual burrows and thus tests our *burrow age hypothesis*, while the *between-burrow age of the breeding wall* effect tests our *burrow quality hypothesis*.

In a separate analysis, we modeled burrow re-occupation probability restricted to the first year following burrow establishment to further explore potential between-burrow effects of the age of the breeding wall. This model included study year as a random intercept effect. We refrained from including in addition a random slope effect for *age of the breeding wall* on study year due to the observed lack of convergence.

Significance of fixed effects was determined by removing the focal term from the current model. *P*-values in the context of GLMM analyses refer to the increase in model deviance when a term is removed from a model compared against a *χ*^2^ distribution using a likelihood ratio test. All GLMM models were fitted in R 3.1.2 (R Core Team, 2013) using the function g*lmer* from the package *lme4* (Bates et al. 2014). All statistical tests were two-tailed and we rejected the null hypothesis at *P* < 0.05.

## Results

Between 2003 and 2013, we recorded a total of 179 European bee-eater broods, of which 97 (54%) took place in a nesting burrow which had already been used in a previous year, indicating that reusing burrows is a common breeding site selection strategy in the bee-eater breeding wall under study. Altogether, 424 burrow-year observations have been recorded for 82 different, individually recognized burrows over the whole observation period (Fig.2). For 342 of the 424 observations and for 75 of the 82 individually recognized burrows, a re-occupation event was possible (the burrows 9 and 25 had existed for a single year only and five burrows first appeared in 2013 which was the last year of the observation period, see Fig.2). In this subsample of 342 observations, individual burrows were frequently re-occupied across years (up to eight times, Fig.2) with re-occupation recorded on 28.4% of the 342 possible occasions. When controlling for pseudoreplication resulting from multiple observations of the same burrows across years as well as multiple observations from the same year using a binomial GLMM, overall burrow re-occupation probability was estimated slightly lower as 26.4% with a 95% confidence interval spanning 10.1% to 53.5%.

**Figure 3:**
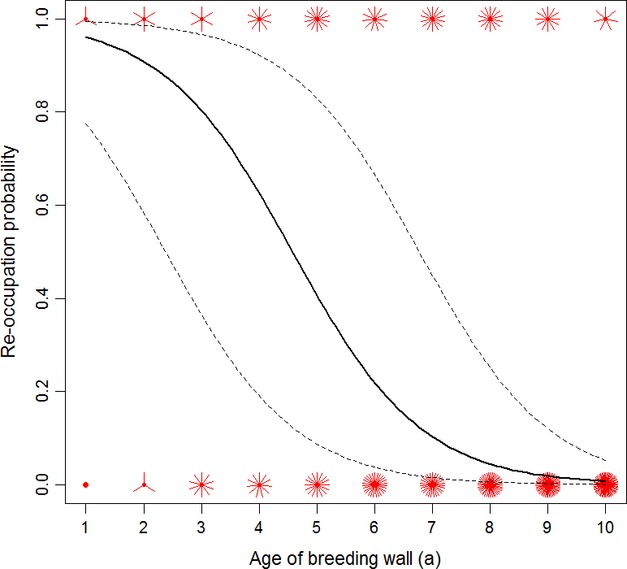
Sunflower plot of the between-year burrow re-occupation probability in a southern German colony of the European bee-eater *Merops apiaster* as a function of the age of the breeding wall (*N* = 342 observations of 75 individually recognized burrows). Sunflower petals indicate number of multiple raw data points, the solid lines show predicted re-occupation probabilities from binomial generalized linear mixed models with burrow identity and study year as random effects, and the dotted lines reflect 95% confidence intervals.

Re-occupation probability showed a highly significant decline with increasing age of the breeding wall (GLMM: *χ*^2^ = 19.0, df = 1, *P* < 0.001; Fig.3; see Table1a for full model representation). However, as not all individual burrows had been monitored across the entire study period, this effect may be due to a decline in re-occupation probability with individual burrow age (*burrow age hypothesis*) or due to a selective disappearance of burrows with relatively high re-occupation probabilities with increasing age of the breeding wall (*burrow quality hypothesis*). Using within-burrow centering of the covariate *age of the breeding wall* to disentangle within- from between-burrow effects, we found that a decline in re-occupation probability of individual burrows with age (*within-burrow age of the breeding wall* effect: *χ*^2^ = 26.6, df = 1, *P* < 0.001), but not selective (dis-)appearance (*between-burrow age of the breeding wall* effect: *χ*^2^ = 0.13, df = 1, *P* = 0.76) was responsible for the observed pattern across time (Figs.3; see Table1b for full model representation). Highly significant random slope variation of the within-burrow *age of the breeding wall* effect (*χ*^2^ = 16.1, df = 2, *P* < 0.001) indicated that individual burrows differed in their re-occupation trajectories with increasing age. To control for the fact that the data are right-hand censored, that is, more data points are available for older than for younger burrows, we restricted the analysis to 316 (of initially 342) observations of 59 (of initially 75) burrows which were first occupied between 2003 and 2010. By excluding those burrows that could be monitored for only 2 or 3 years, we test for the robustness of our analysis. The analyses of the restricted data set yielded very similar results (within-burrow *age of the breeding wall* effect: *χ*^2^ = 26.1, df = 1, *P* < 0.001; between-burrow *age of the breeding wall* effect: *χ*^2^ = 0.46, df = 1, *P* = 0.50; random slope variation of within-burrow *age of the breeding wall* effect: *χ*^2^ = 15.6, df = 2, *P* < 0.001).

**Table 1:** Results from generalized linear mixed models (GLMMs) with binomial error structure and logit link function estimating the effect of the age of the breeding wall on between-year burrow re-occupation probability in a German colony of the European bee-eater *Merops apiaster* (*N* = 342 observations of 75 individually recognized burrows which were occupied for the first time between 2003 and 2012). Note that parameter estimates are given on the logit scale. (a) GLMM including study year and burrow identity as random intercept effects and a random slope effect for *age of the breeding wall*. (b) GLMM including study year and burrow identity as random intercept effects and a random slope effect for the within-burrow predictor of the *age of the breeding wall*. Within-burrow effect: within-burrow effect after within-burrow centering of the focal covariate *age of the breeding wall*. Between-burrow effect: between-burrow effect after within-burrow centering of the focal covariate *age of the breeding wall* (see Methods for details on statistical procedures).

(a)				
Fixed effects	Estimate	SE	*χ* ^2^	*P*
Intercept	−0.82	0.63		
Age of the breeding wall	−8.92	0.23	19.0	<0.001

Random effects	Variance	Correlation		
Burrow identity (intercept)	8.21			
Age of the breedingwall (slope)	0.34	0.23		
Study year (intercept)	0.28			

(b)				
Fixed effects	Estimate	SE	*χ* ^2^	*P*
Intercept	−2.14	0.79		
Within-burrow effect of the age of the breeding wall	−1.20	0.29	26.60	<0.001
Between-burrow effect of the age of the breeding wall	0.09	0.28	0.1	0.76

Random effects	Variance	Correlation		
Burrow identity (intercept)	7.55			
Within-burrow effect of the age of the breeding wall (slope)	0.48	0.43		
Study year (intercept)	0.17			

**Figure 4:**
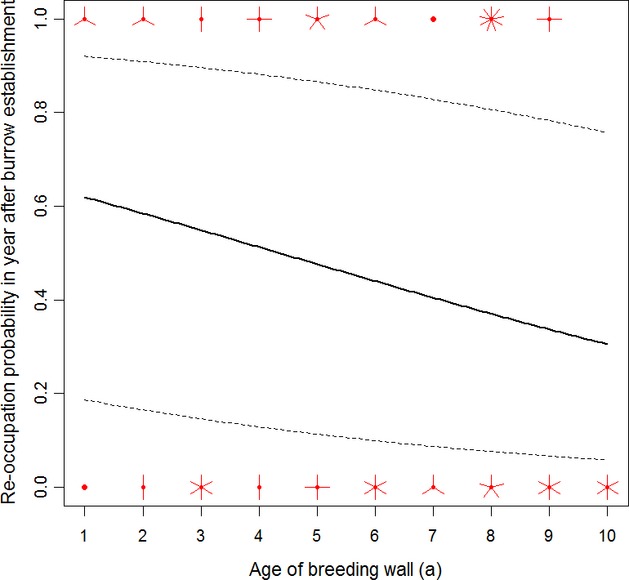
Sunflower plot of the between-year burrow re-occupation probability in a southern German colony of the European bee-eater *Merops apiaster* as a function of the age of the breeding wall restricted to the first year after burrow establishment (*N* = 75 observations of 75 individually recognized burrows). Sunflower petals indicate number of multiple raw data points, the solid lines show predicted re-occupation probabilities from binomial generalized linear mixed models with study year as random effect, and the dotted lines reflect 95% confidence intervals.

Re-occupation probability in the first year following burrow establishment showed no significant relationship with increasing age of the breeding wall (GLMM: *χ*^2^ = 2.0, df = 1, *P* = 0.16; Fig.4; see Table2 for full model representation), indicating that burrows built early on are not of generally higher re-occupation suitability and thus confirming that mainly within-burrow effects are responsible for the general decrease in re-occupation probability with increasing age of the breeding wall. Thus, while the *burrow age hypothesis* is supported by the data, the *burrow quality hypothesis* can be rejected.

**Table 2:** Results from a generalized linear mixed model (GLMM) with binomial error structure and logit link function estimating the effect of the age of the breeding wall on the re-occupation probability of burrows in the year after their establishment (*N* = 75 observations of 75 individually recognized burrows which were occupied for the first time between 2003 and 2012). Study year is included as random intercept effect.

Fixed effects	Estimate	SE	*χ* ^2^	*P*
Intercept	−0.17	0.29		
Age of the breeding wall	−0.15	0.11	2.0	0.16

Random effects	Variance			
Study year (intercept)	0.18			

## Discussion

Our results demonstrate substantial between-year nesting burrow reuse and its temporal dynamics in a European bee-eater colony in southern Germany. More than half of all recorded broods took place in burrows which had been used previously, an estimate that is noteworthily higher than the 14% observed by Rupp and Saumer (1996). For the first time, we also provide a quantitative estimate of burrow reuse probability. The overall probability that a previously used burrow is re-occupied in later years amounted to roughly 26% when statistically controlling for multiple observations of the same burrows across years. Again, this result is in remarkable contrast to the literature record where between-year burrow reuse mostly is described to be absent (Ursprung 1984; Glutz von Blotzheim and Bauer 1994) or very rare at the most (von Erlanger 1900; Fintha 1968; Cramp 1981; Casas-Crivillé and Valera 2005). Our findings may represent a common behavior in the species, as the bee-eaters are not restricted to the wall due to an absence of other suitable breeding walls in the surroundings of our study colony. The investigated bird colony is not restricted to a single breeding wall but started, beginning from the wall under study, to colonize the surrounding area with up to 2013 six occupied breeding walls and 42 breeding pairs in a range from 200 to 2000 m to the founding wall. Nesting opportunities thus are not scarce in the surroundings of the colony under study, and the high rate of burrow reuse that we find consequently does not simply reflect the need to deal with unsuitable surroundings. In conclusion, unless the generally supposed absence of between-year burrow reuse is firmly established in a given breeding wall by means of monitoring individual burrows across years, it seems prudent to assume a decent amount of between-year reuse of existing burrows. As a consequence, digging off parts of a breeding wall containing established burrows – as suggested as breeding wall management for conservation purposes in sand martins (*Riparia riparia*) (Heneberg 2012) and blue-tailed bee-eaters (*Merops philippinus*) (Wang et al. 2009) in part of the literature – should be considered only with utmost caution and rather be avoided in European bee-eaters.

We found that re-occupation probability generally declined with age of the breeding wall and that this effect was attributable solely to individual burrow age (*burrow age hypothesis*). This might simply be due to an elevated risk of collapsing for older burrows which might not always be observable from outside. Additionally, bee-eaters do not normally remove indigestible regurgitates of their offspring or other detritus from their burrows (Krimmer and Piechocki 1974; Ursprung 1984; Glutz von Blotzheim and Bauer 1994). Furthermore, no reports on whether bee-eaters clean established nesting sites before reusing them have been published so far. An accumulation of detritus over the years and an associated higher risk of parasite infection may consequently limit individual burrow reuse suitability with increasing age of the burrow. The trade-off between energy expenditure to build a new burrow and tolerating a higher risk of collapse or an elevated parasite load when reusing an old burrow might thus lead to restricted duty cycles of burrows. Alternatively, burrow re-occupation may presuppose pair fidelity which could also explain the observed restricted burrow reuse duty cycles which would then reflect pair bond durations.

We found highly significant random variation in the re-occupation trajectories between burrows, indicating individual differences in burrow longevity. The previously discussed reasons for restricted reuse cycles of burrows in general can easily be imagined to also cause differences in reuse cycles between individual burrows. For example, burrows may collapse earlier or later depending on the surrounding soil properties which are most likely not homogenous across the breeding wall. Individual differences in the amount of detritus could result from differences in brood size or fledging success. If reuse cycles depend on pair fidelity, the duration of the pair bond would affect an individual burrows duty cycle depending on individual strength of pair bonds and pair survival.

Declining burrow reuse probability over time was not attributable to the selective disappearance of high-quality burrows (*burrow quality hypothesis*). Thus, it seems not to be the case that high-quality burrows with an associated higher reuse probability were built early during colony establishment. Still, the first burrows in newly established breeding walls of burrowing birds are usually built along the upper margin, whereas lower burrows follow only later (Ursprung 1984; Smalley et al. 2013a). While the predation risk arising from beech martens (*Martes foina*) and European badgers (*Meles meles*) is indeed higher when burrows are located in closer proximity to the ground (Sieber 1980; Persson 1987), red foxes (*Vulpes vulpes*) excavate burrows from the top (Heneberg 2005). Besides the risk of predation, especially at the margins of the wall, physical properties such as substrate composition (Smalley et al. 2013b) or rainwater permeability (Smalley et al. 2013a) should play a role in burrow positioning within the wall, too. In the study colony, predation was considered to be very low due to the fact that across the whole study period, nestlings were observed in nearly all active breeding burrows and only one brood loss due to predation was evident (Bastian & Bastian, personal observations). Differences in quality due to a higher predation pressure in later established burrows are therefore unlikely. This fits well to our finding that burrows build in later years seem just as suitable for reuse as those build early on. Given the local conditions with a number of other breeding walls present in close proximity of the study site and the fact that colony growth starts to decline toward the end of our study period with birds starting to disperse to other breeding walls in 2009, birds may just use the wall as long as good positions with regard to physical properties are available and then successively switch to other, new walls in the surrounding area.

Naturally, the present study is limited to a within-colony approach when trying to understand the ecological conditions that promote or hinder nesting burrow reuse in European bee-eaters. It would therefore be worthwhile to extend the approach taken here over the breeding range of the species as, for example, the availability and quality of breeding walls and the demographic and genetic composition of populations may well differ, especially in the margin regions of the distribution. Based on quantitative data from many different populations, such an extension will promote a better understanding of the ecology and ultimately conservation of a European flagship species for conservation and for studying population responses to environmental change.
